# Evaluation of Reliability and Validity of the Hendrich II Fall Risk Model in a Chinese Hospital Population

**DOI:** 10.1371/journal.pone.0142395

**Published:** 2015-11-06

**Authors:** Congcong Zhang, Xinjuan Wu, Songbai Lin, Zhaoxia Jia, Jing Cao

**Affiliations:** 1 International Medical Services, Peking Union Medical College Hospital, Beijing, 100730, China; 2 Nursing Department, Peking Union Medical College Hospital, Beijing, 100730, China; 3 Beijing Obstetrics and Gynecology Hospital, Capital Medical University, Beijing Maternal and Child Health Care Hospital, Beijing, 100026, China; Cardiff University, UNITED KINGDOM

## Abstract

To translate, validate and examine the reliability and validity of a Chinese version of the Hendrich II Fall risk Model (HFRM) in predicting falls in elderly inpatient. A sample of 989 Chinese elderly inpatients was recruited upon admission at the Peking Union Medical College Hospital. The inpatients were assessed for fall risk using the Chinese version of the HFRM at admission. The reliability of the Chinese version of the HFRM was determined using the internal consistency and test-rested methods. Validity was determined using construct validity and convergent validity. Receiver operating characteristic (ROC) curves were created to determine the sensitivity and specificity. The Chinese version of the HFRM showed excellent repeatability with an intra-class correlation coefficient (ICC) of 0.9950 (95% confidence interval (CI): 0.9923–0.9984). The inter-rater reliability was high with an ICC of 0.9950 (95%CI: 0.9923–0.9984). Cronbach’s alpha coefficient was 0.366. Content validity was excellent, with a content validity ratio of 0.9333. The Chinese version of the HFRM had a sensitivity of 72% and a specificity of 69% when using a cut-off of 5 points on the scale. The area under the curve (AUC) was 0.815 (P<0.001). The Chinese version of the HFRM showed good reliability and validity in assessing the risk of fall in Chinese elderly inpatients.

## Introduction

Falls are described as any sudden, unexpected and unintentional occurrence resulting in a patient landing on the ground or at lower level [1]. Prevention of patient falls is an important aspect of patient safety management, particularly in elderly patients who often present impaired balance and who are at higher risk of fracture [2]. In patients ≥65 years old, at least one fall will occur within a year in about 30% of community dwellers and in 50% of patients in nursing homes [3]. In the USA, the overall 2-year prevalence of >1 falls in patients over 65 years was 36.3% [4]. The most fatal injuries in the elderly are from falls [3].

Therefore, tools are necessary to assess the risk of falls to improve the safety of patients in the hospital. The St. Thomas risk assessment tool in falling elderly inpatients (STRATIFY) tool assesses five factors with good sensitivity (93%) and specificity (88%) [5], but does not appear to be useful in patients undergoing geriatric rehabilitation [6]. The Morse fall score (MFS) is also used, but is has shown lower sensitivity (76%) and specificity (68%) compared with the STRATIFY tool [7]. The Hendrich II Fall Risk Model (HFRM) is a scale used to evaluate the risk of patient falls in American hospitals [8]. This tool was developed specifically for the risk assessment of patient falls in hospital and the scale needs only 3–5 minutes to complete [9].

In China, patient falls prevention is also considered as a crucial component of hospital patient safety management. However, no tool is currently available in Chinese to evaluate the risk of falls of elderly inpatients in China. Since the HFRM is easy to use, the present study aimed to translate, validate and examine the reliability and validity of a Chinese version of the HFRM tool in predicting falls in elderly inpatients.

## Methods

### Subjects

This was a cross-sectional study performed from August 2013 to July 2014. The participants were recruited from seven departments in the Peking Union Medical College Hospital (neurology, endocrinology, nephrology, infectious diseases, respiratory medicine, Chinese medicine and geriatric wards). Consecutive patients were selected to represent the Chinese elder inpatients population. Inclusion criteria were: 1) aged ≥60 years; and 2) hospitalized for chronic diseases. Exclusion criteria were: 1) being hospitalized ≤3 days; or 2) being hospitalized for critical illness and being bedridden.

The present study was approved by the ethical committee of the Peking Union Medical College Hospital. Written informed consent was obtained from each patient.

### Data collection

After obtaining the consent of the author (Dr. Ann Hendrich), the study group translated the HFRM tool into Chinese. The instrument consists of eight variables: confusion/disorientation/impulsivity (No = 0, Yes = 4); symptomatic depression (No = 0, Yes = 2); altered elimination (No = 0, Yes = 1); dizziness vertigo (No = 0, Yes = 1); gender (female = 0, male = 1); any administered anti-epileptics (No = 0, Yes = 2); any administered benzodiazepines (No = 0, Yes = 1); "get up and go" test (0–4). Patients were considered at high risk of falling when they scored >5 [10]. Researchers with a special training for using the Chinese version of the HFRM tool evaluated the risk of falling of all participants within 24 hours after hospitalization. After the first evaluation, all participants were evaluated every week using the same instrument.

A questionnaire was designed to collect participants’ demographic characteristics, admission diagnosis, presence of chronic diseases, the number of days of hospitalization, if the patient was accompanied during hospitalization, if the patient ever fell during hospitalization, and if the patient used assistive devices (crutches, wheelchair, etc.). Whether the patients fell or not was determined by nursing documentation or by patients’ reporting.

A convenience sample of 120 participants from the original 989 was recruited in order to determine the test-retest and inter-rater reliability. Sixty cases were evaluated again by the same researcher 3 days after the first evaluation. The other 60 patients was recruited in order to determine the inter-rater reliability. Two researchers evaluated the same subjects independently. Validity was determined by a panel group composed of six experts who have worked at the hospital for >20 years.

### Statistical analysis

Continuous variables are expressed as mean±SD or median (interquartile range), as appropriate. Categorical data are expressed as frequencies. Cronbach's α coefficient was calculated to evaluate the internal consistency reliability of the Chinese version of the HFRM tool. Intraclass correlation coefficient (ICC) was used to assess the test-retest and inter-rater reliability. A factor analysis was used to examine the construct validity of the instrument using the Kaiser-Meyer-Olkin measure of sampling adequacy and Bartlett’s test of sphericity. Finally, receiving operator characteristic (ROC) curve analysis was performed to determine sensitivity and specificity and to evaluate the predictive validity. SPSS 12.0 (SPSS Inc., Chicago, IL, USA) was used for statistical analyses. All tests were two-sided and P<0.05 was considered statistically significant.

## Results

### Characteristics of the subjects

A total of 989 subjects were enrolled in this study (497 male and 492 female). Age ranged from 60 to 92 years, for a mean of 66.0±6.9 years. The shortest hospitalization was 4 days, and the longest was 80 days (mean of 19.8±10.5 days). About 72.6% of the subjects had a poor vision, 36.4% suffered from hearing loss, and 5% used crutches, wheelchairs or other mobility aids. In this study, falls occurred for32 patients during hospitalization. Only one patient had leg bruising; there was no injury in the remaining 31 participants. (Table 1)

**Table 1:** Baseline characteristics of study subjects.

	Number	Percentage (%)
Gender		
Male	497	50.25
Female	492	49.75
Vision		
Normal	271	27.40
Poor	718	72.60
Hearing		
Normal	629	63.60
Loss	360	36.40
Accompanied		
Yes	460	46.51
No	529	53.49
Mobility aids (crutches, wheelchairs)		
Yes	49	5.00
No	940	95.00
Falls history		
Yes	389	39.33
No	600	60.67
Injury caused by falls before this hospitalization		
Yes	79	7.99
No	910	92.01
With chronic diseases		
Yes	890	89.99
No	90	10.01
Falls during this hospitalization		
Yes	32	3.24
No	957	96.79
Injury caused by falls during this hospitalization		
Yes	1	0.10
No	988	99.90

### Reliability of the Chinese version of the HFRM

The test-retest reliability, inter-rater reliability and internal consistency reliability were assessed. The intra-class correlation coefficient was 0.9950 (95%CI: 0.9923–0.9984). The intra-class correlation coefficient (ICC) of inter-rater reliability was 0.9950 (95% CI: 0.9923–0.9984). The Cronbach’s α was 0.336.

### Validity of the Chinese version of the HFRM

The panel group agreed that all items of the HFRM were relevant to detect the likelihood of falls in at-risk patients. However, they suggested modifying some language expressions to make the scale more concise and easier to understand. For example, “Discharge changes” instead of “Abnormal discharge”. The panel group also evaluated the content validity of HFRM. The content validity ratio (CVR) was calculated and the average CVR was 0.9333. The Kaiser-Meyer-Olkin (KMO) measure of sampling adequacy was 0.543, and Bartlett’s test of sphericity was 366.802 (*P*<0.001). The items were found loading into three factors, and the cumulative percentage accounted for 50.117% of the total variance using principal component analysis. The Eigenvalues of the three components were 1.571, 1.220, and 1.218, and accounted for 19.638%, 15.251%, and 15.228% of the total variance respectively.

**Fig 1:**
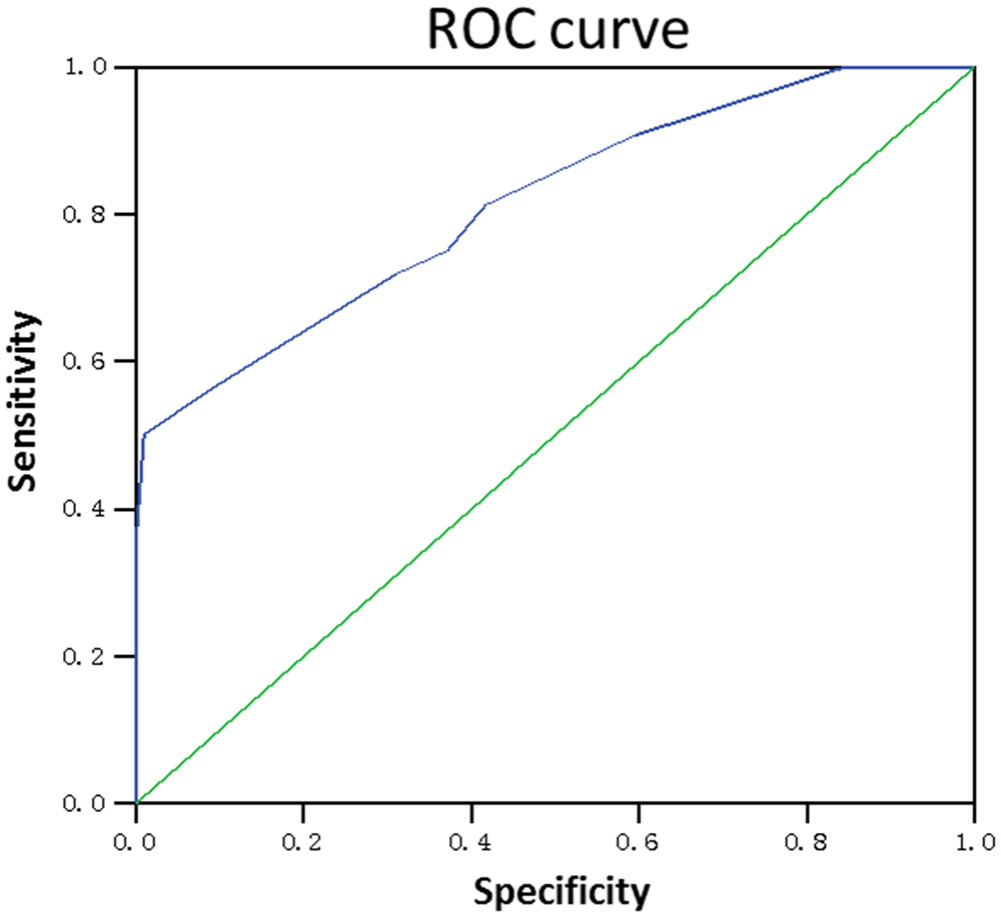
ROC curve analysis of the Chinese version of the HFRM in predicting falls in Chinese elderly inpatients.

The predictive validity of HFRM was tested for sensitivity, specificity, positive predictive value and negative predictive value. Falls occurred for32 participants during hospitalization. The HFRM correctly predicted 23 falls, for a sensitivity of 72%, a specificity of 69%, a positive predictive value of 7%, and a negative predictive value of 98% (Table 2). ROC analyses indicated that the area under the curve (AUC) was 0.815 (*P*<0.001) (Fig 1).

**Table 2:** Cross table of HFRM prediction of falls and actual falls during hospitalization.

	Falls	
	Yes	No
HFRM score		
High risk	23	297
Low risk	9	660

## Discussion

The aim of the present study was to translate, validate and examine the reliability and validity of a Chinese version of the Hendrich II Fall risk Model (HFRM) in predicting patient falls. Results showed that the Chinese version of the HFRM had excellent repeatability, as shown by excellent ICC of 0.9950 and inter-rater ICC of 0.9950. Cronbach’s alpha coefficient was 0.366. Content validity was excellent. The Chinese version of the HFRM had a sensitivity of 72% and a specificity of 69% when using a cut-off of 5 points on the scale. The AUC was 0.815.

Healthcare staff, even within hospitals, can only combine their practical experience and the patients’ actual situation to obtain some subjective guidance to prevent falls from happening. However, falls still happen. There is no complete system or tool to assess, prevent and intervene falls in China [11]. In recent years, accidental falls happening to hospitalized patients has gradually gained people's attention. Falls have negative impacts such as delaying patient rehabilitation, suing, dissatisfaction and economic losses. Therefore, early assessment and identification of risk factors is an effective premise to prevention. Patients at high risk of falls can be effectively identified using appropriate predictive tools [7].

In this study, the Crobach's alpha coefficient of the Chinese version of the HFRM was 0.336. Usually, a Cronbach’s alpha ≥0.7 is considered as good internal consistency reliability. Generally, scales with more items will have higher Crobach's alpha coefficient. However, it is possible to add items to enhance the Crobach's alpha coefficient, but the scale becomes more complicated. Hospital medical workers have no time to lose in using long and complicated scales [12]. Therefore, even though the scale had a low Crobach's alpha coefficient, it might be more welcome by the hospital medical workers because of its simplicity.

There are many scales for assessing fall risk including STRATIFY, HFRM,MFS, Tinetti, Downtown and Tullamore [7]. The STRATIFY, HFRM and MFS tools are relatively mature scales compared with the other scales, and they have been compared together in some studies [7]. Indeed, a study had compared the reliability and validity of these three scales in an Australia hospital using 5489 cases, and every case was assessed using these three scales; finally, the HFRM had the best balance of sensitivity and specificity, but STRATIFY required the less time to complete [13]. Therefore, we wanted to introduce HFRM in China to improve the identification of patients at high risk of falling.

HFRM has been adapted to different cultures in the world. In 2013, Nassar et al. assessed the predictive value of two instruments (MFS and HFRM) in a Middle Eastern country. The results showed that the HFRM was more sensitive in predicting falls than the MFS [14]. In 2007, Kim et al. evaluated the validity of three fall-risk assessment tools to identify patients at high risk for falls (MFS, STRATIFY and HFRM); the HFRM had a more acceptable specificity (61.5%) [13]. In 2013, Caldevilla et al. adapted and evaluated the HFRM for use in elderly Portuguese inpatients, and showed a sensitivity of 93.2% and 75.7%, and a specificity of 35% and 46.7%, upon admission and at discharge, respectively, with positive predictive values of 17.2% and 17.0%, and negative predictive values of 97.3% and 93%, respectively [15]. In 2011, Ivziku et al. evaluated the predictive validity and inter-rater reliability of HFRM; the inter-rater reliability was 0.87 (95% CI 0.71–1.00). Sensitivity and specificity were 86% and 43%. The optimal cut-off score for screening at risk patients was 5 with an area under the ROC curve of 0.72. These results provided supporting evidence for the choice of the HFRM to screen older patients at risk of falling in acute care settings [16], and supported the results of the present study.

The present study is not without limitations. First, all participants were recruited from one hospital; thus, the results might not be extrapolated to the general Chinese elderly population. Secondly, a too short test-retest interval might increase the test-retest reliability measures. Finally, although this Chinese version of the HFRM showed good reliability and validity in assessing the fall risk of elderly inpatients, other scale translated in Chinese may also be good. Future, studies might be performed using different scales in different Chinese elderly populations.

In conclusion, the Chinese version of the HFRM had good reliability and validity in assessing the fall risk of elderly inpatients. However, comparisons with other tools should be performed.

## Supporting Information

S1 Raw Data (DOCX)

S1 Questionnaire Questionnaire in Chinese version. (DOCX)
