# Supplementary material for: Evaluation of Reliability and Validity of the Hendrich II Fall Risk Model in a Chinese Hospital Population
Source: PLoS One. 2015 Nov 6;10(11):e0142395. doi: 10.1371/journal.pone.0142395 (PMC4636230; doi:10.1371/journal.pone.0142395)

**1、三个量表的比较：**

| 项目 MFS STRATIFY Hendrich II Fall risk Model |
| --- |
| 临界值 ≥25 ≥2 ≥5  敏感度 88.3% 55% 70%  特异度 48.3% 75.3% 61.5%  +PV(95%CI) 1.9(1.4-2.4) 2.4(1.7-3.4) 2.0(1.5-2.6)  -PV(95%CI) 99.7(99.5-99.9) 99.3(99.0-99.5) 99.5(99.2-99.7)  AUC(95%CI) 80(73-86) 71(65-77) 73(67-80)  Kappa值 0.86 0.87 0.87 |

**2、研究对象的一般资料：**

| 项目 | 人数（例） 百分比（%） |
| --- | --- |
| 性别  男 497 50.25  女 492 49.75  视力  正常 271 27.40  欠佳或老花 718 72.60  听力  正常 629 63.60  下降 360 36.40  陪护  无陪护 529 53.49  有陪护 460 46.51  助行器（拐杖、轮椅等）  使用助行器 49 5.00  未使用助行器 940 95.00  跌倒史  有跌倒史 389 39.33  无跌倒史 600 60.67  既往跌倒是否引起损伤  有损伤 79 7.99  无损伤 910 92.01  有无第二诊断  有第二诊断 969 97.98  无第二诊断 20 2.02  有无慢性病  有慢性病 890 89.99  无慢性病 90 10.01  住院期间是否发生跌倒  跌倒 32 3.24  未跌倒 957 96.76  住院期间跌倒是否引起损伤  有损伤 1 0.10  无损伤 988 99.90 | |

**3、两次测量结果的组内相关系数：**

| 总分 | N $\overline{x}$±S | ICC | 95%CI |
| --- | --- | --- | --- |
| 全量表总分 | 60 2.90±2.24 | 0.9950 | 0.9923,0.9984 |

两名评估者测量结果的组内相关系数：

| 总分 | N $\overline{x}$±S | ICC | 95%CI |
| --- | --- | --- | --- |
| 全量表总分 | 60 2.88±2.241 | 0.9950 | 0.9923,0.9984 |

**4、内容效度比：**

CVR=$\frac{n-N/2}{N/2}$

公式中n为专家中认为条目与测定内容很相关的人数，N为专家总数。本研究各条目的内容效度比具体如下^[66]^：

条目l(意识模糊) ………………………… 6-6／2/6/2=1.0000

条目2(抑郁) …………………………… 6-6／2/6/2=1.0000

条目3(排泄方式改变) ………………… 6-6／2/6/2=1.0000

条目4(头晕) ……………………………… 6-6／2/6/2=1.0000

条目5(男性) ……………………………… 5-6／2/6/2=0.6667

条目6(服用抗癫痫药物) ………………… 6-6／2/6/2=1.0000

条目7(服用苯二氮晫类药物) …………… 6-6／2/6/2=1.0000

条目8(起立-行走测试) ……………………6-6／2/6/2=1.0000

**5、KMO抽样适度测定值与Bartlett球形检验值：**

| 项目 |  | 分值 |
| --- | --- | --- |
| KMO抽样适度测定值  Bartlett球形检验：近似  卡方值  P自由度 |  | 0.543  366.802  28  0.000 |

**6、各成分的特征值及方差贡献率：**

| 成分 | 特征值 | 方差贡献率 | 累计方差贡献率 |
| --- | --- | --- | --- |
| 1 | 1.571 | 19.638 | 19.638 |
| 2 | 1.220 | 15.251 | 34.889 |
| 3 | 1.218 | 15.228 | 50.117 |
| 4 | 0.978 | 12.224 | 62.342 |
| 5 | 0.898 | 11.222 | 73.563 |
| 6 | 0.826 | 10.324 | 83.888 |
| 7 | 0.714 | 8.924 | 92.812 |
| 8 | 0.575 | 7.188 | 100.000 |

**7、碎石图**

**8、旋转后因子成分矩阵：**

| 条目 因子1 因子2 因子3 |
| --- |
| 意识障碍/定向力障碍/行为冲动  **0.636**  0.122 0.114  抑郁状态  **0.809**  0.000 0.061  排泄方式改变 -0.073  **0.654** 0.017  头晕/眩晕 -0.017 -0.157  **0.590**  男性 0.129  **0.663** -0.394  服用抗癫痫药  **0.689** -0.114 -0.050  服用苯二氮晫类药物 0.122 0.008  **0.605**  起立-行走测试 0.031 0.547 **0.574** |

**9、2×2四格表：**

| 跌倒 未跌倒 |
| --- |
| (量表评估)有风险 23 （a） 297 （b）  (量表评估)无风险 9 （c） 660 （d） |

敏感度Sn=a(a+c)=23÷(23+9)=72%；

特异度Sp=d(b+d)=660÷(297+660)=69%；

阳性预测率+PV=a/(a+b)=23÷320=7%；

阴性预测率-PV=d/(c+d)=660÷669=98%；

**10、Hendrich跌倒风险评估量表筛查住院老年患者的ROC曲线下面积：**

| 曲线下面积 | 标准误 | P | 95% 可信区间 |
| --- | --- | --- | --- |
| 0.815 | 0.043 | 0.000 | 0.730，0.900 |

**11、Hendrich跌倒风险评估量表各分值所对应的敏感度和特异度：**

| 分值 | 敏感度 | 1-特异度 | 特异度 | 诊断指数DI | Yunden指数 |
| --- | --- | --- | --- | --- | --- |
| -1.00  .50  1.50  2.50  3.50  4.50  5.50  6.50  7.50  8.50  9.50  11.00  12.50  13.50  15.00 | 1.000  1.000  0.906  0.813  0.750  **0.719**  0.563  0.500  0.375  0.281  0.156  0.094  0.063  0.031  0.000 | 1.000  0.854  0.594  0.417  0.371  0.310  0.090  0.009  0.001  0.001  0.001  0.000  0.000  0.000  0.000 | 0.000  0.155  0.406  0.583  0.628  **0.690**  0.910  0.991  0.999  0.999  0.999  1.000  1.000  1.000  1.000 | 1.000  1.155  1.312  1.396  1.378  1.409  1.473  1.491  1.374  1.280  1.155  1.094  1.063  1.031  1.000 | 0.000  0.000  0.312  0.396  0.122  0.409  0.473  0.491  0.374  0.280  0.155  0.094  0.063  0.031  0.000 |

**12、 病人一般情况调查表**

**《病人一般情况调查表》**

病人姓名： 性别：

年龄： 科室：

入院诊断：

第二诊断：

住院天数：

视力： 正常 欠佳

听力： 正常 欠佳

是否有陪护：是 否

使用助行器：是 否

慢性病： 有 无

跌倒史： 有 无 ；跌倒后有无损伤：

住院期间是否发生跌倒： 是 否

住院期间跌倒后有无损伤：有 无

**13、Hendrich II Fall Risk Model（原英文量表）**


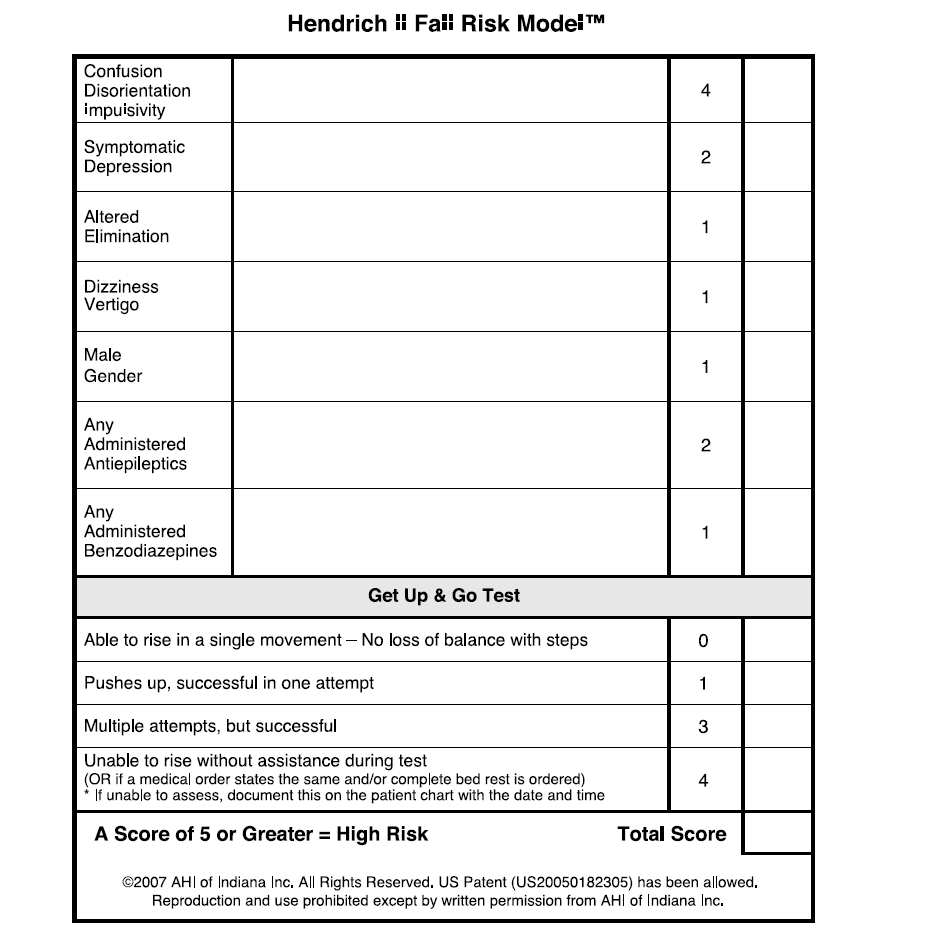

Supplement: S1 Raw Data — (DOCX) [file pone.0142395.s001.docx]
