# Supplementary material for: Evaluation of Reliability and Validity of the Hendrich II Fall Risk Model in a Chinese Hospital Population
Source: PLoS One. 2015 Nov 6;10(11):e0142395. doi: 10.1371/journal.pone.0142395 (PMC4636230; doi:10.1371/journal.pone.0142395)
Supplement: S1 Questionnaire — (DOCX) [file pone.0142395.s002.docx]

**《病人一般情况调查表》**

病人姓名： 性别：

年龄： 科室：

入院诊断：

第二诊断：

住院天数：

视力： 正常 欠佳

听力： 正常 欠佳

是否有陪护：是 否

使用助行器：是 否

慢性病： 有 无

跌倒史： 有 无 ；跌倒后有无损伤：

住院期间是否发生跌倒： 是 否

住院期间跌倒后有无损伤：有 无
